# Supplementary material for: Mapping QTLs for Breast Muscle Weight in an F2 Intercross between Native Japanese Nagoya and White Plymouth Rock Chicken Breeds
Source: Life (Basel). 2021 Jul 27;11(8):754. doi: 10.3390/life11080754 (PMC8399377; doi:10.3390/life11080754)
Supplement: Supplementary file 1 [file life-11-00754-s001.zip › life-1296169-supplementary.pdf]

Supplementary Materials

Mapping QTL for breast muscle weight in an F_2 intercross between native Japanese Nagoya and White Plymouth Rock chicken breeds

Ishikawa, A. et al.

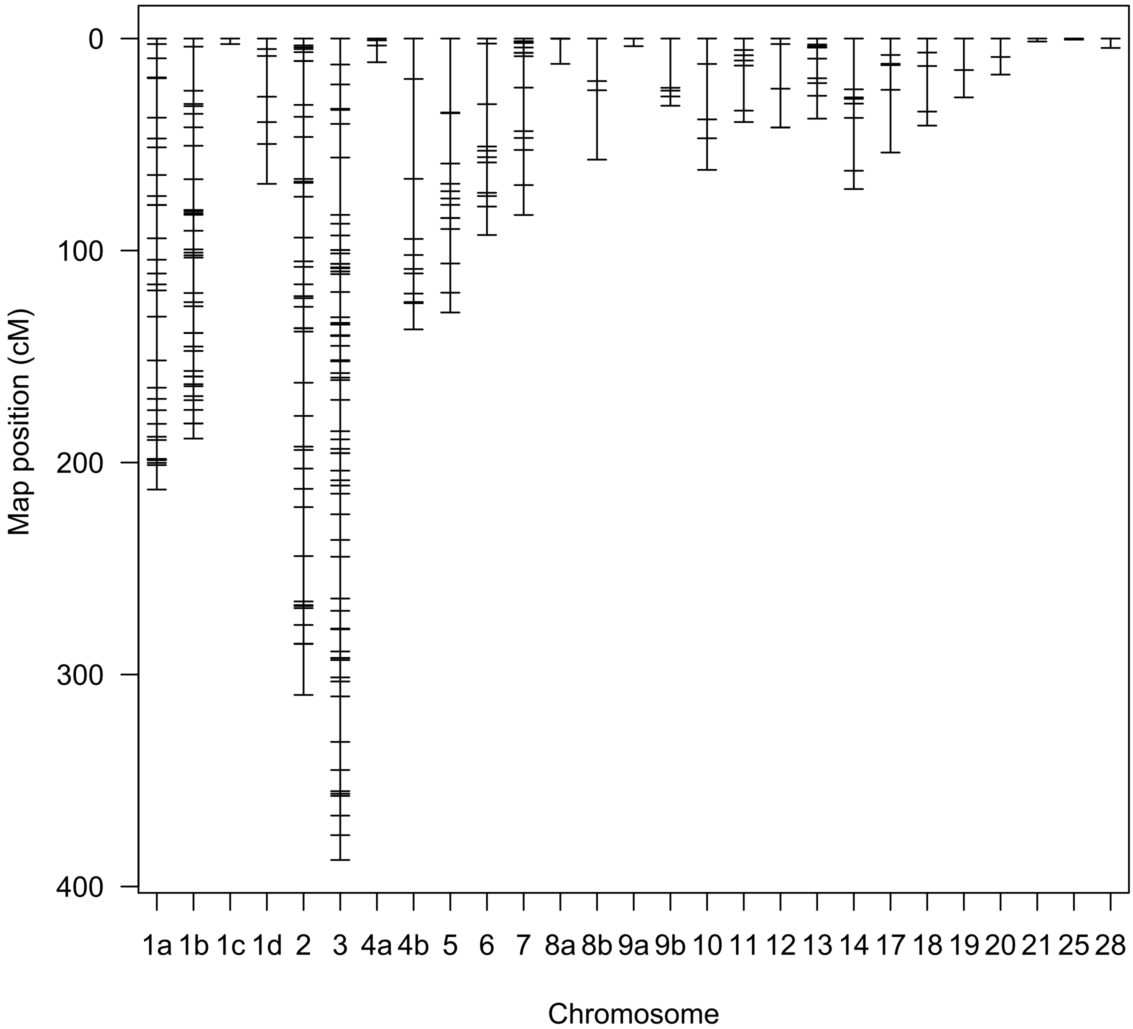

Figure S1. The linkage map of 313 SNP markers constructed by our previous study [16]. Short horizontal lines show the map positions of the SNP markers which were assigned to 27 linkage groups on 21 autosomes. Chromosome 1 was divided into four linkage groups (1a-1d), and chromosomes 4, 8 and 9 were each divided into two linkage groups (4a, 4b, 8a, 8b, 9a and 9b).

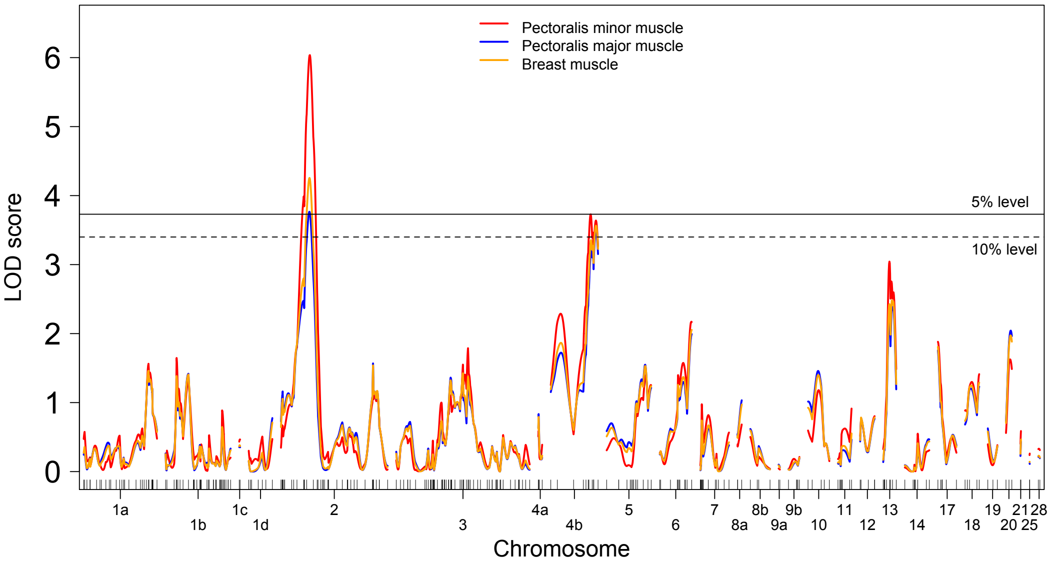

Figure S2. Genome-wide LOD score plots obtained by single-QTL genome scans for three breast muscle weights. The weight of breast muscle is the sum of weights of pectoralis minor muscle and pectoralis major muscle. See Table S1 for details of genome-wide 10% and 5% significant threshold levels.

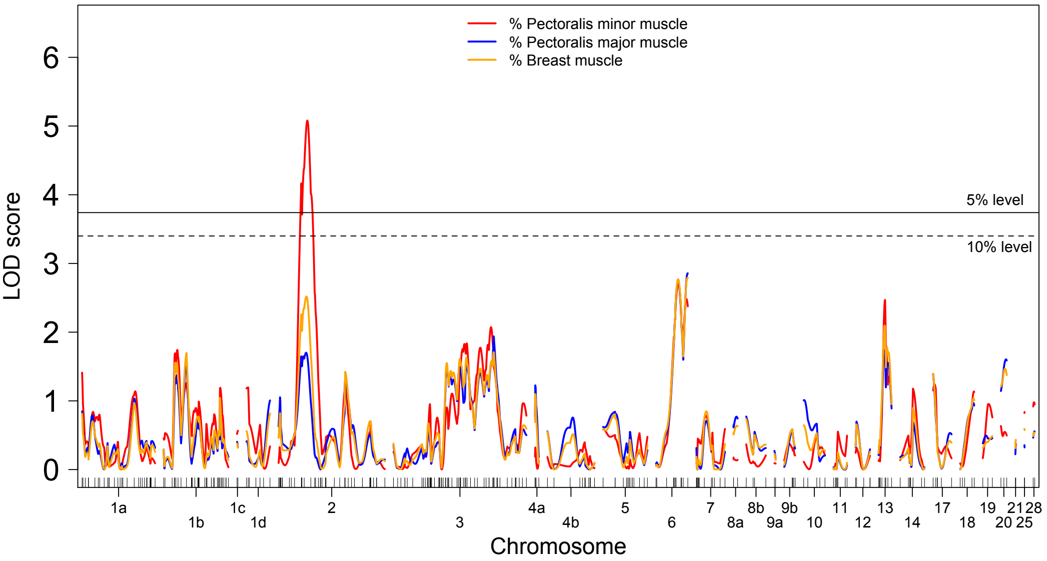

Figure S3. Genome-wide LOD score plots obtained by single-QTL genome scans for percentages of three breast muscle weights to body weight at 4 weeks of age.

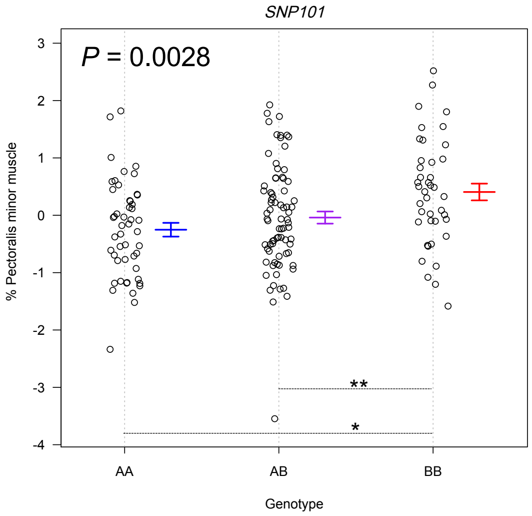

Figure S4. Genotype effect plots of the *SNP101* marker nearest to the QTL for the percentage of pectoralis minor weight to body weight at 4 weeks of age. Each circle shows muscle weight in SD unit for an animal. Horizontal bars for each genotype show mean ± SE. The *P* value was obtained from one-way ANOVA. $^*P < 0.05$ and $^{**}P < 0.01$ refer to statistical differences between two genotypes (Tukey's honestly significant difference test). A: NAG allele, B: WPR allele.

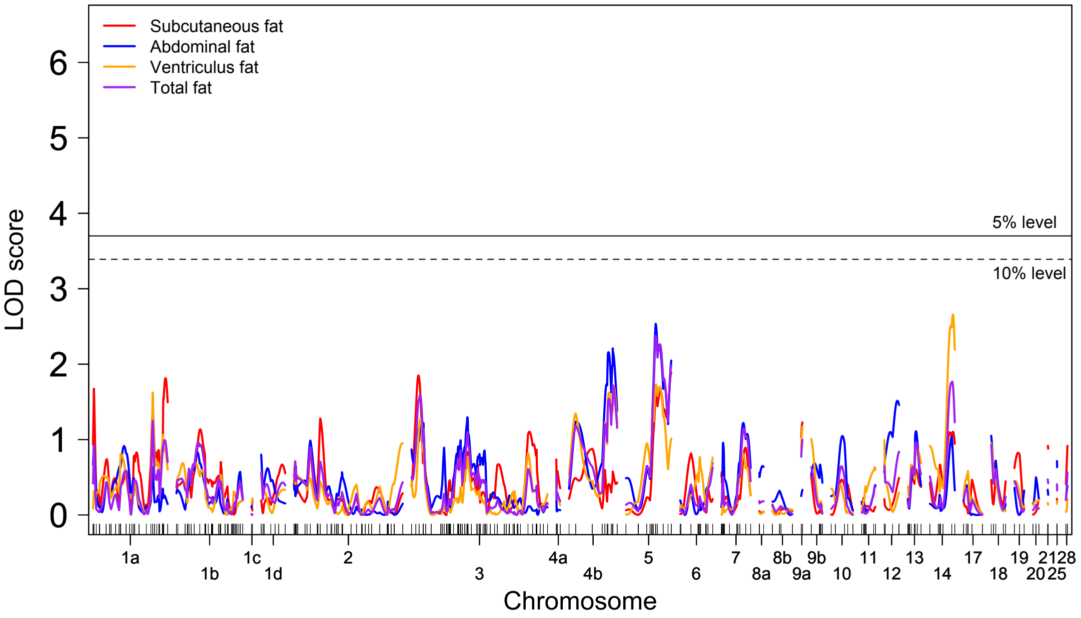

Figure S5. Genome-wide LOD score plots obtained by single-QTL genome scans for fat weights. Total fat weight is the sum of weights of subcutaneous, abdominal and ventriculus fats.

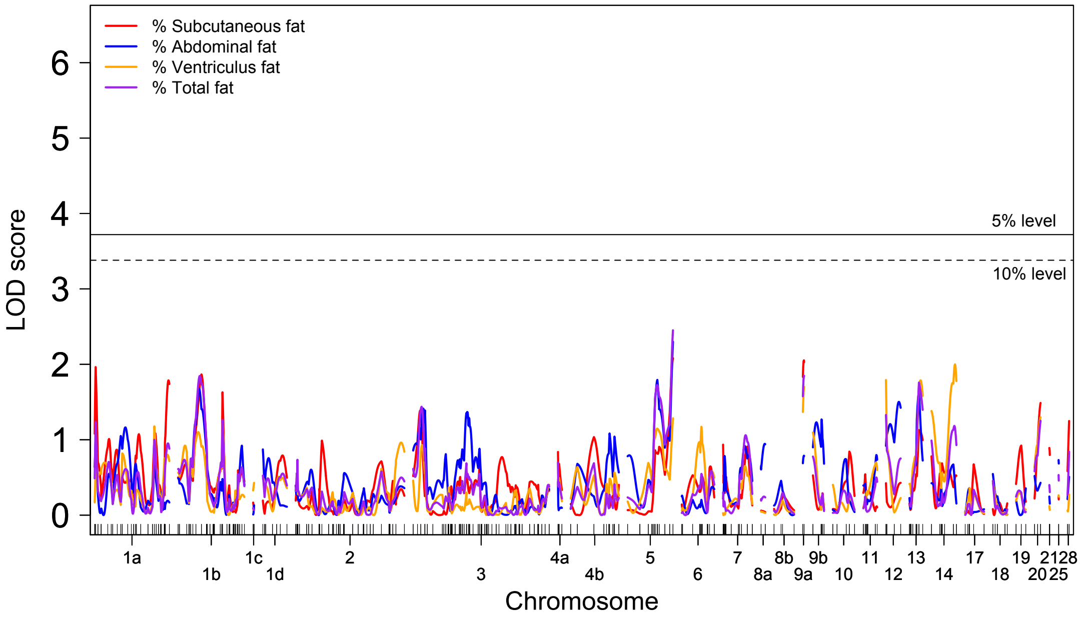

Figure S6. Genome-wide LOD score plots obtained by single-QTL genome scans for percentages of fat weights to body weight at 4 weeks of age.

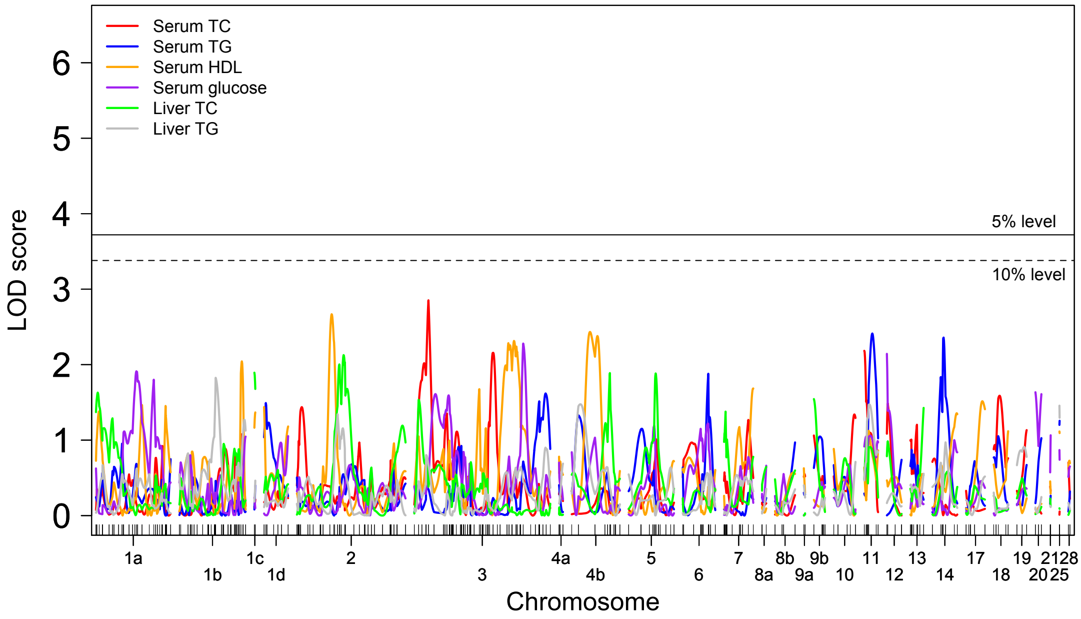

Figure S7. Genome-wide LOD score plots obtained by single-QTL genome scans for serum and liver levels of biochemical parameters. TC: total cholesterol, TG: triglycerides, HDL: high density lipoprotein.

Table S1. Genome-wide significance threshold levels used for single-QTL genome scans.

Trait	1%	5%	10%
Pectoralis minor muscle	4.44	3.70	3.37
Pectoralis major muscle	4.49	3.72	3.38
Breast muscle	4.51	3.77	3.40
% Pectoralis minor muscle	4.46	3.72	3.38
% Pectoralis major muscle	4.50	3.74	3.40
% Breast muscle	4.54	3.74	3.40
Abdominal fat	4.46	3.68	3.38
Ventriculus fat	4.46	3.69	3.40
Subcutaneous fat	4.50	3.74	3.39
Total fat	4.44	3.72	3.37
% Abdominal fat	4.58	3.77	3.39
% Ventriculus fat	4.47	3.71	3.38
% Subcutaneous fat	4.46	3.68	3.35
% Total fat	4.43	3.70	3.38
Serum TC	4.45	3.69	3.37
Serum TG	4.46	3.71	3.37
Serum HDL	4.51	3.74	3.38
Serum glucose	4.52	3.73	3.40
Liver TC	4.45	3.72	3.39
Liver TG	4.45	3.75	3.38
